# Comparative analyses of genetic trends and prospects for selection against hip and elbow dysplasia in 15 UK dog breeds

**DOI:** 10.1186/1471-2156-14-16

**Published:** 2013-03-02

**Authors:** Thomas W Lewis, Sarah C Blott, John A Woolliams

**Affiliations:** 1 Kennel Club Genetics Centre at the Animal Health Trust, Lanwades Park, Kentford, Newmarket, Suffolk CB8 7UU, UK; 2 The Roslin Institute and Royal (Dick) School of Veterinary Studies, University of Edinburgh, Easter Bush Research Centre, Midlothian EH25 9RG, UK

**Keywords:** Canine, Hip dysplasia, Elbow dysplasia, Estimated breeding value, Selection, Accuracy, Genetic correlation, Heritability, Welfare

## Abstract

**Background:**

Hip dysplasia remains one of the most serious hereditary diseases occurring in dogs despite long-standing evaluation schemes designed to aid selection for healthy joints. Many researchers have recommended the use of estimated breeding values (EBV) to improve the rate of genetic progress from selection against hip and elbow dysplasia (another common developmental orthopaedic disorder), but few have empirically quantified the benefits of their use. This study aimed to both determine recent genetic trends in hip and elbow dysplasia, and evaluate the potential improvements in response to selection that publication of EBV for such diseases would provide, across a wide range of pure-bred dog breeds.

**Results:**

The genetic trend with respect to hip and elbow condition due to phenotypic selection had improved in all breeds, except the Siberian Husky. However, derived selection intensities are extremely weak, equivalent to excluding less than a maximum of 18% of the highest risk animals from breeding. EBV for hip and elbow score were predicted to be on average between 1.16 and 1.34 times more accurate than selection on individual or both parental phenotypes. Additionally, compared to the proportion of juvenile animals with both parental phenotypes, the proportion with EBV of a greater accuracy than selection on such phenotypes increased by up to 3-fold for hip score and up to 13-fold for elbow score.

**Conclusions:**

EBV are shown to be both more accurate and abundant than phenotype, providing more reliable information on the genetic risk of disease for a greater proportion of the population. Because the accuracy of selection is directly related to genetic progress, use of EBV can be expected to benefit selection for the improvement of canine health and welfare. Public availability of EBV for hip score for the fifteen breeds included in this study will provide information on the genetic risk of disease in nearly a third of all dogs annually registered by the UK Kennel Club, with in excess of a quarter having an EBV for elbow score as well.

## Background

Hip dysplasia may be described as one of the most serious hereditary diseases occurring in pedigree dogs given the popularity of susceptible breeds and the prevalence therein [1,2]. It is also one of the most persistent, first having been described over 50 years ago [3-5]. Hip dysplasia is a developmental orthopaedic disorder characterised by the formation of a dysmorphic, lax (loose) coxo-femoral (hip) joint [6]. Over time, particularly in larger and giant breeds, the malformation and laxity lead to the abnormal wearing of bone surfaces and the appearance of the osteoarthritic signs of degenerative joint disease (DJD) [7]. The resultant osteoarthritis (OA) is irreversible and so the only way to effect a lasting and widespread improvement in the welfare of susceptible breeds is through genetic selection. Hip dysplasia remains a significant problem, despite the presence of several evaluation schemes across the world designed to provide an empirical phenotype for selection, partly due to its complexity; a polygenic background and multiple environmental influences ensure no clear pattern of inheritance. Furthermore, the breeding guidelines accompanying evaluation schemes have often elicited only very weak selection [8,9].

In contrast elbow dysplasia, despite also being a developmental orthopaedic abnormality long recognised as a serious problem [10], has historically received less attention than hip dysplasia. As a result, schemes evaluating elbow condition are younger than those examining hips, and so data is less abundant. The term ‘elbow dysplasia’ commonly describes a number of abnormalities associated with developmental physiological incongruity of the elbow joint that often result in OA [11].

This grouping of syndromes for both the pathology and evaluation of elbow dysplasia may result in under-estimates of heritability [12]; which range from 0.10 to 0.38 [13-17] among various breeds. Analyses of more specific elbow abnormalities have estimated higher heritabilities; for example 0.57 for fragmented coronoid process in German Shepherd Dogs [9]. Estimates of heritability of hip condition generally have a smaller range but appear moderate in magnitude, from 0.20 to 0.43 across various breeds [8,14,16,18-20] despite using data from different international scoring schemes and hips being evaluated on both detectable laxity and OA. The reported genetic correlation between hip and elbow condition varies even more, from −0.09 to 0.42 [9,14,16,17].

Many recent studies estimating the genetic parameters of hip and elbow dysplasia score data have recommended selection using estimated breeding values (EBV; [8,9,14,16,19-21]. EBV are the best linear unbiased predictor (BLUP) of every dog’s breeding value derived from the pedigree information used in its calculation [1], and are a more accurate estimate of the genetic liability of a trait than the individual phenotype. However, attempts to quantify the potential benefit to the response to selection against hip and elbow dysplasia that the increased accuracy of selection using EBV would bring (compared to phenotypic selection) are less common than parameter estimation, but have been made empirically by Lewis et al. [8], and via simulation by Stock and Distl [22] and Malm et al. [23]. Improvements in the rate of genetic progress (which is directly related to the accuracy of selection, [24]) would be achieved not only through EBV acting as a more accurate predicator of genetic risk (i.e. the true breeding value) than phenotype, but also through enhanced opportunities to increase selection intensity due to EBV being available for every dog in the pedigree [25]. EBV would effectively provide a greater quantity of more reliable information with respect to breeding. This study, therefore, aims to estimate the genetic parameters of hip and elbow dysplasia in the UK registered breeds for which score data is most abundant, determine any genetic trends and evaluate potential improvements in response to selection due to increased accuracy and abundance of reliable information that publication of EBV would provide.

## Methods

### Data

Phenotype data comprised results of the British Veterinary Association (BVA)/UK Kennel Club (KC) hip and elbow scoring schemes. Details of scoring protocols are given by Gibbs [26] and Lewis et al. [17]. In brief, radiographs of hips are scored bilaterally on 9 features according to the degree of laxity and/or OA observed (8 features scored 0 to 6, one feature scored 0 to 5). The aggregate of the 18 scores reported ranges from 0 (indicating no malformation) to 106 (severe hip dysplasia). The BVA/KC elbow scoring scheme was launched in 1998 based on guidelines of the International Elbow Working Group (IEWG). Elbow radiographs are scored according to the size of detectable primary lesions and severity and extent of OA observed, ranging from 0 (normal) to 3 (severe elbow dysplasia). The score of the worst elbow only is publically reported. Pedigree data was provided by the KC and linked to phenotype data via a unique registration number.

Fifteen breeds (Akita [AKT], Bearded Collie [BEARD], Bernese Mountain Dog [BMD], Border Collie [BORD], English Setter [ENG], Flat Coat Retriever [FCR], Gordon Setter [GDN], Golden Retriever [GR], German Shepherd Dog [GSD], Labrador Retriever [LAB], Newfoundland [NEWF], Rottweiler [ROTT], Rhodesian Ridgeback [RR], Siberian Husky [SHUSK] and Tibetan Terrier [TT]) were included in the study. For 5 breeds (BMD, GR, GSD, LAB and ROTT) the genetic parameters of hip and elbow score were estimated using bivariate analyses. For the remaining 10 breeds, the genetic parameters of hip score only were estimated using univariate analyses.

For the ten breeds with hip score only, genetic parameters and EBV were estimated simultaneously using data from dogs evaluated at 365–1459 days old and between 1990 and 2011 inclusive, and the entire KC electronically recorded pedigree extending back to the early 1980s; hip score having undergone transformation to improve normality (see below). For BMD and ROTT genetic parameters and EBV were computed simultaneously for hip and elbow data via bivariate REML analyses using evaluations from dogs of the same age and study period and the entire KC electronic pedigree. The pedigrees of LAB, GSD and GR were too large to include in their entirety in bivariate parameter estimation on a desktop PC, and so for parameter estimation in these breeds data and/or depth of pedigree was truncated. For GSD and GR genetic parameters of hip and elbow score were estimated using data from all dogs of the same age and study period with a further 5 generations of pedigree. For LAB genetic parameters of hip and elbow scores were estimated using data from all dogs evaluated at the same age and between 2000–2011, and 2 further generations of pedigree. The genetic parameters for LAB, GSD and GR were then used in the calculation of BLUP EBV using hip and elbow data from 1990–2011 and the entire KC pedigrees of each breed (GR pedigree = 386,580 animals; GSD pedigree = 572,552 animals; LAB data = 59,077 evaluations, pedigree = 977,083 animals), undertaken by Edinburgh Genetic Evaluation Service (EGENES) using MiX99. The numbers of records used in the REML analyses of hip score for each breed are shown in Additional file 1: Table S1.

Thus, data for EBV computation included 142,287 hip scores from all fifteen breeds, which have a total mean of 82,118 registrations per year (2000 to 2010 data), and 13,908 elbow scores from BMD, GR, GSD, LAB and ROTT; these breeds having a total mean of 70,363 registrations per year (2000–2010 data).

### Analyses

Mixed linear models were fitted using ASREML [27]. For univariate analysis of hip score the model used was as per Lewis et al. (2010) [8]. For bivariate analysis of hip and elbow score the model used was as per Lewis et al. (2011) [17].

Total hip score was log transformed (after adding 1 to avoid necessitating the logarithm of zero) to improve normality. Where applicable the untransformed mean of left and right elbow score was included as a y-variate. The possible transformation of observed values to more closely correspond to the underlying liability [17] was not undertaken as the benefits were found to be small and because, importantly, the transformation depends on the prevalence which may change over time. Data from 3 year old animals (1095–1459 days) were included for consistency with hip data and after preliminary analysis using Labrador data showed the genetic correlation of elbow score at 365–1094 days and 1095–1495 days (i.e. 1–2 and 3 year olds) was indistinguishable from 1.

The general form of the univariate linear model was as follows:

Y=Xb+Za+Wc+e

where **Y** is the vector of observations, **W**, **X** and **Z** are known incidence matrices, **b** is the vector of fixed effects; **a** is the vector of random additive genetic effects with the distribution assumed to be multivariate normal (MVN), with parameters (0, σ^2^_a_**A**); **c** is the vector of random litter effects with the distribution assumed to be MVN, with parameters (0, σ^2^_c_**I**_**litter**_), and **e** is the vector of residuals distributed MVN with parameters (0, σ^2^_e_**I**). **I** represents an identity matrix of an appropriate size, **A** is the additive genetic relationship matrix and σ^2^ denotes the variance of each of the respective random effects. To extend this univariate model to bivariate analyses the variance terms such as σ^2^_a_ were replaced by the appropriate bivariate covariance matrices (Σ) for the traits using the Kronecker product, such as *A* ⊗ *Σ*_*A*_. The phenotypic variance is denoted as σ^2^_P_, and heritability (*h*^*2*^) is calculated as the proportion of phenotypic variance explained by the additive genetic variance (σ^2^_A_/σ^2^_P_). Phenotypic, additive genetic and residual correlations (r_P_, r_A_, r_E_) were computed from the genetic (co)variances obtained.

Fixed effects included in the model were: sex, inbreeding coefficient (as calculated using the entire KC electronic pedigree), age in days at evaluation, absolute day of birth (measured as days since 1st January 1980) and year of evaluation. Age in days and absolute day of birth were fitted with random smoothing splines to model temporal trends [8].

### Meta-analysis of parameter estimates across breeds

The spread of parameter estimates will be due to two components: (i) sampling errors within a breed, and (ii) variation in the true parameter among breeds. A meta-analysis of the parameter estimates was undertaken to obtain the best estimate of the mean parameter for the population of breeds, together with a standard error to account for both sampling and population variation. This followed the procedures of Corbin et al. [28]. The analysis provides an estimate of the variance of the true parameter among breeds, and if this is 0 then the pooled mean is identical to that obtained from using a weight for each breed equal to the reciprocal of its sampling variance.

### Accuracy of estimated breeding values

The accuracy (*r*) of each animal’s EBV was calculated as:

$$r=\sqrt{1-\frac{\mathrm{PEV}}{\left(1+F\right)\sigma_{A}^{2}}}$$

(see Additional file 2), where PEV is the prediction error variance of each EBV, F is the inbreeding coefficient for each animal and σ^2^_A_ is the estimated additive genetic variance obtained from the mixed model analysis. ASREML provides both the estimates of the EBV and their associated PEVs.

Potential advantages of using EBV in future selection for lower hip/elbow scores were evaluated by comparison of mean EBV accuracies with the predicted accuracy of phenotypic selection in all breeds. Firstly, the mean EBV accuracy of phenotyped animals born in 2010 (with no progeny phenotypes) was compared to the accuracy of phenotypic selection (h, [24]). Secondly, mean accuracy of EBV for animals born in 2011 (<365 days old and therefore without a phenotype), but for which both parental phenotypes were available, was compared to the accuracy of selection using these phenotypes (√(½).h, see Additional file 3) to determine any potential improvement in the response to selection of breeding animals prior to obtaining their own scores. Finally, the proportion of animals born in 2011 (so without a phenotype) with EBV accuracy exceeding √(½).h was calculated and compared to the proportion where both parental phenotypes were available.

### Assessment of genetic gain to date

The genetic gain as a proportion of genetic standard deviation was calculated as: (mean EBV_maxyr_-mean EBV_minyr_)/ σ_A_. For hip score *minyr* = 1990, and for elbow score *minyr* = 2000; *maxyr* = 2011 for both traits. The trends in genetic disposition to hip/elbow score were discerned for each breed via regression of EBVs on date of birth, and intensity of selection (*i*) applied estimated by rearrangement of the following equation:

$$\mathrm{\Delta G}=ih^{2}\sigma_{P}/L$$

where *ΔG* is the genetic trend determined by regression of EBV on date of birth, *h*^*2*^ is the heritability, *σ*_*P*_ is the phenotypic standard deviation, and *L* is the generation interval.

## Results

### Hips

**Figure 1:**
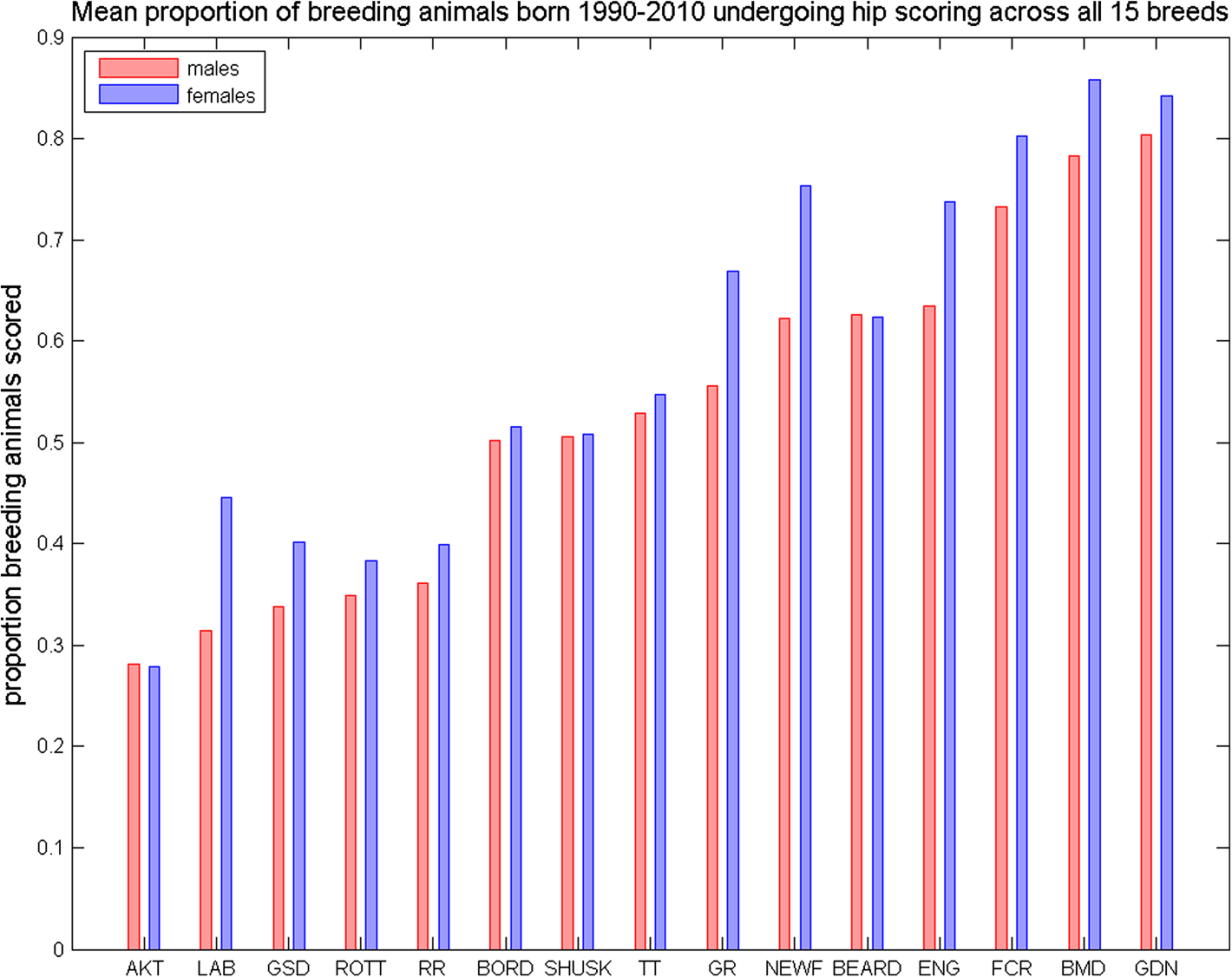
**Average proportion of breeding animals hip scored.** Mean proportion of male and female breeding animals born annually from 1990–2010 that are hip scored for all 15 breeds.

**Figure 2:**
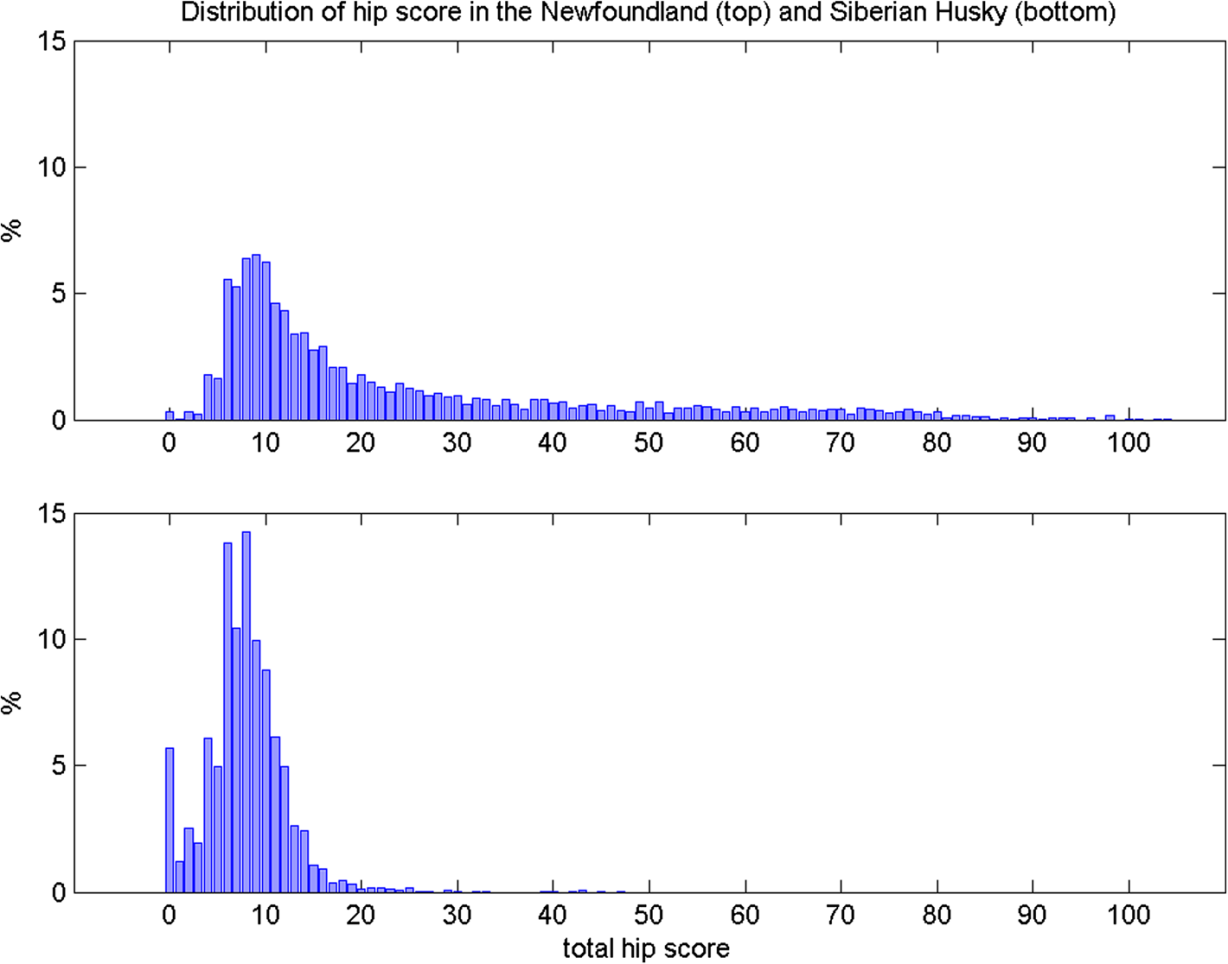
**Hip score distribution for Newfoundland and Siberian Husky.** Distribution of total hip score for the Newfoundland (top) and Siberian Husky (bottom) breeds, from dogs evaluated between 1990–2011 and 365–1459 days old.

An average of between 6% (GSD) and 19% (GDN) of all dogs registered annually since 1990 had been hip scored. The rate of scoring is higher for breeding animals, with the mean percentage of breeding animals born annually since 1990 having undergone hip scoring ranging from 27% of sires and 28% of dams (AKT) to 80% of sires (GDN) and 86% of dams (BMD), Figure 1. There was considerable variation in the distribution of total untransformed hip scores (Figure 2 and Additional file 1: Table S1), with mean hip score ranging from 7.89 (SHUSK) to 23.35 (NEWF), mode from 6 to 10, median from 8 to 14, and standard deviation from 4.38 (SHUSK) to 20.49 (NEWF). All distributions were highly skewed, with coefficient of skewness ranging from 1.46 (NEWF) to 4.59 (FCR), reflecting the cumulative nature of the scoring system [29].

The results of the analyses determined that the BEARD displayed the smallest phenotypic variation (0.219) in log transformed total hip score and the NEWF the largest (0.605, Table 1). The FCR exhibited the smallest degree of additive genetic variation (0.073) of log transformed total hip score and the NEWF the largest (0.279). Estimates of heritability of log transformed total hip score ranged from 0.28 (FCR) to 0.48 (SHUSK). Estimates of litter variance as a proportion of phenotypic variance (not shown) ranged from 0.017 (AKT) to 0.141 (GDN), although litter was not a significant effect in all models. Meta-analysis of estimates of heritability of hip score across the 15 breeds indicated only a small degree of heterogeneity among breeds, with a mean estimate of heritability across breeds of 0.38 (s. e. 0.014). The estimate of variance of between breed heritability estimates was 1.8 x 10^-3^.

**Table 1:** Parameter estimates of hip score

**Breed**	**σ**^**2**^_**P**_	**σ**^**2**^_**A**_	**h**^**2**^	**s.e.**
AKT	0.478	0.187	0.39	0.053
BEARD	0.219	0.100	0.46	0.048
BORD	0.223	0.098	0.44	0.033
ENG	0.295	0.104	0.35	0.049
FCR	0.257	0.073	0.28	0.032
GDN	0.450	0.194	0.43	0.062
NEWF	0.605	0.279	0.46	0.041
RR	0.445	0.146	0.33	0.048
SHUSK	0.349	0.167	0.48	0.038
TT	0.246	0.084	0.34	0.048
BMD	0.355	0.129	0.36	0.040
GR	0.313	0.126	0.40	0.017
GSD	0.390	0.138	0.35	0.015
LAB	0.381	0.126	0.33	0.012
ROTT	0.308	0.120	0.39	0.028

Estimates of phenotypic and additive genetic variance (σ^2^_P_and σ^2^_A_ respectively) and heritability (h^2^, with standard error) of hip score for 15 breeds. The top panel shows parameters for 10 breeds derived from univariate analyses, while the bottom panel shows parameters for 5 breeds derived from bivariate analyses of hip and elbow score. Breed abbreviations: Akita [AKT], Bearded Collie [BEARD], Bernese Mountain Dog [BMD], Border Collie [BORD], English Setter [ENG], Flat Coat Retriever [FCR], Gordon Setter [GDN], Golden Retriever [GR], German Shepherd Dog [GSD], Labrador Retriever [LAB], Newfoundland [NEWF], Rottweiler [ROTT], Rhodesian Ridgeback [RR], Siberian Husky [SHUSK] and Tibetan Terrier [TT].

Regression of EBV on date of birth showed recent improving (negative) genetic trends significantly different to zero (P < 0.01) in all cases except that of the SHUSK, where the genetic disposition towards higher (unfavourable) hip score, while still determinable (P < 0.01), increased at a rate of 0.8% per year (Table 2). Those breeds showing an improving genetic trend ranged from a decline in genetic propensity toward hip score of −0.13% per year (FCR) to −1.98% per year (NEWF) on the untransformed scale. However, of those breeds showing an improving genetic trend the derived selection intensities are weak; equivalent to excluding between less than 2% (BEARD, FCR and RR) and less than 18% (GDN) of the highest risk animals from breeding. As a result the genetic progress made has been slow, with the difference in mean EBV from animals born in 1990 and 2011 equating to between only 0.12 (BEARD) and 0.82 (NEWF and GDN) of respective genetic standard deviations.

**Table 2:** Estimates of genetic progress and selection pressure for hip and elbow score

**Hips**	**Progress / σ**_**A**_	***b (x10***^***-2***^***)***	***i***	**p excluded**
AKT	−0.28	−0.66	−0.08	<0.04
BEARD	−0.12	−0.16	−0.04	<0.02
BORD	−0.36	−0.63	−0.13	<0.07
ENG	−0.67	−1.07	−0.24	<0.13
FCR	−0.17	−0.13	−0.04	<0.02
GDN	−0.82	−1.95	−0.32	<0.18
NEWF	−0.82	−2.00	−0.22	<0.12
RR	−0.19	−0.32	−0.06	<0.02
SHUSK	0.25	0.81	0.12	N/A
TT	−0.36	−0.52	−0.13	<0.06
BMD	−0.30	−0.68	−0.12	<0.06
GR	−0.71	−1.20	−0.23	<0.13
GSD	−0.48	−0.89	−0.16	<0.08
LAB	−0.77	−1.28	−0.28	<0.16
ROTT	−0.59	−0.78	−0.14	<0.07
**Elbows**				
BMD	−0.20	−0.72	−0.11	<0.06
GR	−0.13	−0.31	−0.09	<0.04
GSD	−0.14	−0.21	−0.14	<0.07
LAB	−0.13	−0.18	−0.12	<0.06
ROTT	−0.21	−0.39	−0.15	<0.08

Genetic progress was estimated in two ways: total change as mean EBV_2011_-mean EBV_1990_ (for hips, EBV_2011_-mean EBV_2000_ for elbows) as proportion of genetic standard deviation (σ_A_) and annually by the regression coefficient (*b*) of EBV on date of birth. Selection pressure was described in two ways: standardised selection intensity (*i*) against hip/elbow score, and the equivalent proportion of breeding individuals excluded required to achieve that intensity by truncation of the distribution. The top panel shows parameters for 10 breeds derived from univariate analyses of hip score and the middle panel shows hip parameters for 5 breeds derived from bivariate analyses of hip and elbow score. The bottom panel shows elbow parameters for 5 breeds derived from bivariate analyses of hip and elbow score. Breed abbreviations: Akita [AKT], Bearded Collie [BEARD], Bernese Mountain Dog [BMD], Border Collie [BORD], English Setter [ENG], Flat Coat Retriever [FCR], Gordon Setter [GDN], Golden Retriever [GR], German Shepherd Dog [GSD], Labrador Retriever [LAB], Newfoundland [NEWF], Rottweiler [ROTT], Rhodesian Ridgeback [RR], Siberian Husky [SHUSK] and Tibetan Terrier [TT].

The mean accuracies of EBV of phenotyped animals born in 2010 were higher than the predicted accuracy of selection on phenotype (h) for all breeds, ranging from an improvement of 8% (BEARD and SHUSK) to 24% (FCR) (Table 3). The mean accuracies of un-phenotyped animals born in 2011 but with phenotyped parents were higher than the anticipated accuracy of selection on parental phenotypes for all breeds by between 18% (SHUSK) and 47% (ENG). Importantly the anticipated accuracy of selection on parental phenotypes (√(½).h) is optimistic since it ignores all potential biases from fixed effects and changes in the addititive genetic variance over generations due to selection [30]. The proportion of all animals registered in 2011 with EBV accuracies greater than that anticipated from selection on parental phenotypes was always greater than the proportion of animals for which such phenotypes were actually available. The increment ranged from 2% (from 92.8% with both parental phenotypes to 94.8% with EBV accuracy > √½.h; ENG), to an increase of over three-fold (from 19.5% with both parental phenotypes to 59.7% with EBV accuracy > √½.h; AKT). In some cases this jump was not particularly large, ENG and GDN for example have increments of just 2% and 6% respectively, but in these cases the increment in actual (mean) EBV accuracy compared to √½.h is large (47% and 32% respectively).

**Table 3:** Increment in accuracy of selection for low hip score using EBV versus phenotype

	**Animals with phenotype**				**Animals with parental phenotype**				**Proportion with r > √½.h**		
	**h**	**mean r**	**n**	**incr**	**√½.h**	**mean r**	**n**	**incr**	**EBV**	**pheno**	**incr**
AKT	0.62	0.74	23	1.18	0.44	0.64	129	1.45	0.597	0.195	3.05
BEARD	0.68	0.73	26	1.08	0.48	0.61	324	1.29	0.923	0.806	1.15
BORD	0.66	0.74	98	1.11	0.47	0.58	910	1.23	0.745	0.583	1.28
ENG	0.59	0.72	18	1.21	0.42	0.62	180	1.47	0.948	0.928	1.02
FCR	0.53	0.66	54	1.24	0.38	0.53	1067	1.39	0.998	0.866	1.15
GDN	0.66	0.73	30	1.12	0.46	0.61	145	1.32	0.928	0.873	1.06
NEWF	0.68	0.75	37	1.11	0.48	0.58	441	1.21	0.829	0.697	1.19
RR	0.57	0.66	20	1.16	0.40	0.52	521	1.28	0.844	0.502	1.68
SHUSK	0.69	0.74	12	1.08	0.49	0.58	288	1.18	0.478	0.209	2.29
TT	0.58	0.70	45	1.19	0.41	0.55	712	1.34	0.928	0.736	1.26
BMD	0.60	0.71	48	1.19	0.43	0.56	402	1.31	0.893	0.754	1.18
GR	0.63	0.73	277	1.15	0.45	0.54	5097	1.21	0.860	0.791	1.09
GSD	0.59	0.69	337	1.15	0.42	0.51	3343	1.21	0.571	0.441	1.29
LAB	0.57	0.70	1004	1.21	0.41	0.52	16160	1.28	0.685	0.494	1.39
ROTT	0.63	0.73	51	1.17	0.44	0.59	565	1.34	0.568	0.361	1.57
**Mean**				**1.16**				**1.30**			**1.44**

(Left panel) The mean accuracy (r) of EBV of phenotyped animals born in 2010 compared to accuracy of phenotypic selection (h), with the sample size (n) and increment in accuracy (incr). (Middle panel) The mean accuracy of EBV of unphenotyped animals born in 2011, but with parental phenotypes, compared to the accuracy of selection on parental phenotypes (√(½).h). (Right panel) The proportion of unphenotyped animals born in 2011 with EBV accuracy exceeding √(½).h (EBV) compared to the proportion of 2011 born animals with parental phenotypes available (pheno). The top panel utilised parameters for 10 breeds derived from univariate analyses, while the bottom panel utilised parameters for 5 breeds derived from bivariate analyses of hip and elbow score. Increments calculated prior to rounding. Breed abbreviations: Akita [AKT], Bearded Collie [BEARD], Bernese Mountain Dog [BMD], Border Collie [BORD], English Setter [ENG], Flat Coat Retriever [FCR], Gordon Setter [GDN], Golden Retriever [GR], German Shepherd Dog [GSD], Labrador Retriever [LAB], Newfoundland [NEWF], Rottweiler [ROTT], Rhodesian Ridgeback [RR], Siberian Husky [SHUSK] and Tibetan Terrier [TT].

### Elbows

Since 2000 between 1% (GR, GSD, LAB) and 15% (BMD) of all registered dogs of the 5 relevant breeds have been elbow scored. The rate of scoring is higher for breeding animals, with the mean percentage of breeding animals born annually since 2000 having undergone elbow scoring ranging from 8% of sires and 7% of dams (ROTT) to 66% of sires and 77% of dams (BMD). There was variation in the distribution of untransformed elbow scores with mean elbow score ranging from 0.15 (LAB) to 0.61 (ROTT), standard deviation from 0.46 (LAB) to 0.87 (BMD) and coefficient of skewness from 0.92 (ROTT) to 3.59 (LAB) (Additional file 4: Table S2). The LAB displayed the smallest phenotypic variation (0.196) and additive genetic variation (0.037) in elbow score and the BMD the largest (0.760 and 0.201 respectively). Estimates of heritability of untransformed mean elbow score ranged from 0.14 (ROTT) to 0.30 (GR) (Table 4). Meta-analysis of estimates of heritability of elbow score across the 5 breeds indicated only a small degree of heterogeneity, with an across-breed estimate of heritability of 0.218 (s.e. 0.026). The estimate of variance of between breed heritability estimates was similar to but smaller than that for hip score at 0.8 x 10^-3^. Estimates of litter variance as a proportion of phenotypic variance (not shown) ranged from 0.007 (BMD) to 0.146 (ROTT), although litter was not a significant effect in all models. The genetic correlation between hip and elbow scores ranged from 0.005 (BMD) to 0.550 (ROTT). However, the genetic correlation between the two traits was only determinable as significantly different from zero in LAB (P < 0.001). The deviation of the correlation from zero in ROTT approached significance (P = 0.055), suggesting that more data may have increased the power to detect significance. Meta-analysis of estimates of genetic correlation between hip and elbow score across the 5 breeds indicated a greater degree of heterogeneity among breeds than found with the heritabilities, with an across-breed estimate of genetic correlation of 0.216 (s.e. 0.076). The estimate of variance of between breed genetic correlation estimates was 13.1 x 10^-3^.

**Table 4:** Parameter estimates of elbow score

	**σ**^**2**^_**P**_	**σ**^**2**^_**A**_	**h**^**2**^	**s.e.**	**r**_**A**_	**s.e.**	**r**_**E**_	**s.e.**
BMD	0.760	0.201	0.26	0.054	0.005	0.134	0.122	0.051
GR	0.278	0.084	0.30	0.054	0.137	0.098	0.095	0.050
GSD	0.265	0.048	0.18	0.062	0.203	0.140	−0.054	0.055
LAB	0.196	0.037	0.19	0.028	0.344	0.064	−0.003	0.024
ROTT	0.533	0.073	0.14	0.106	0.550	0.299	−0.091	0.091

Estimates of phenotypic and genetic variance (σ^2^_P_and σ^2^_A_ respectively) and heritability (h^2^) of elbow score and genetic and residual correlations (r_A_ and r_E_ respectively, with standard errors) with hip score for 5 breeds. Breed abbreviations: Bernese Mountain Dog [BMD], Golden Retriever [GR], German Shepherd Dog [GSD], Labrador Retriever [LAB], Rottweiler [ROTT].

Regression of EBV on date of birth showed a recent slow but significantly (P < 0.05) improving genetic trend (Table 2) in all 5 breeds, ranging from a decline in genetic propensity toward elbow score of between −0.18% per year (LAB) to −0.72% per year (BMD). The derived selection intensities were very weak; equivalent to excluding between only less than 4-8% of the highest risk animals from breeding. As a result the genetic progress made has been slow, with the difference in mean EBV from animals born in 2000 and 2011 equating to between only 0.13 (LAB) and 0.21 (ROTT) of respective genetic standard deviations.

The mean accuracies of EBV of phenotyped animals born in 2010 were higher than the predicted accuracy of selection on phenotype (h) for all breeds, ranging from an improvement of 17% (GR) to 52% (ROTT) (Table 5). The mean accuracies of un-phenotyped animals born in 2011 but with phenotyped parents were similarly greater than the anticipated accuracy of selection on parental phenotypes by between 23% (GR) and 71% (ROTT). The proportion of all animals registered in 2011 with EBV accuracies greater than that anticipated from selection on parental phenotypes was greater than the proportion of animals for which both parental phenotypes were actually available in all 5 breeds, the increment ranging from 23% (from 72.2% with both parental phenotypes to 88.9% with EBV accuracy > √½.h; BMD) to a greater than 10-fold increase (from 6% with both parental phenotypes to 79.5% with EBV accuracy > √½.h; ROTT).

**Table 5:** Increment in accuracy of selection for low elbow score using EBV versus phenotype

	**Animals with phenotype**				**Animals with parental phenotype**				**Proportion with r > √½ h**		
	**h**	**mean r**	**n**	**incr**	**√½ h**	**mean r**	**n**	**incr**	**EBV**	**pheno**	**incr**
BMD	0.51	0.66	46	1.28	0.36	0.52	385	1.43	0.889	0.722	1.23
GR	0.55	0.64	136	1.17	0.39	0.48	959	1.23	0.385	0.149	2.59
GSD	0.42	0.51	197	1.21	0.30	0.38	535	1.26	0.272	0.071	3.85
LAB	0.43	0.59	579	1.37	0.31	0.45	3411	1.45	0.600	0.104	5.76
ROTT	0.37	0.56	28	1.52	0.26	0.45	95	1.71	0.795	0.061	13.09
**Mean**				**1.23**				**1.34**			**3.14**

(Left panel) The mean accuracy (r) of EBV of phenotyped animals born in 2010 compared to accuracy of phenotypic selection (h), with the sample size (n) and increment in accuracy (incr). (Middle panel) The mean accuracy of EBV of unphenotyped animals born in 2011, but with parental phenotypes, compared to the accuracy of selection on parental phenotypes (√(½).h). (Right panel) The proportion of unphenotyped animals born in 2011 with EBV accuracy exceeding √(½).h (EBV) compared to the proportion of 2011 born animals with parental phenotypes available (pheno). Increments calculated prior to rounding. Breed abbreviations: Bernese Mountain Dog [BMD], Golden Retriever [GR], German Shepherd Dog [GSD], Labrador Retriever [LAB], Rottweiler [ROTT].

### The effect of inbreeding

The effects of inbreeding coefficient were typically very small and not significantly different to zero in all cases, except on hip score in the RR (−0.69, s.e. = 0.350) and on elbow score in the GR (0.83, s.e. = 0.316). In the RR this corresponds to a decline of 0.75 points for the median hip score of 8 (or a 4.24 point decrease from a hip score of 50) comparing coefficient of inbreeding of 0.125 to 0 (values obtained for offspring of a half-sib and unrelated matings respectively). In the GR there is an increase of 0.1 points in elbow score comparing coefficient of inbreeding of 0.125 to 0.

## Discussion

The results from this study demonstrate the potential power of EBV to improve the predicted accuracy of selection against hip and elbow dysplasia in many dog breeds in the UK, including 3 of the 10 most popular breeds. The mean accuracies of EBV are always higher than would be obtained via selection on available phenotypes (using either individual or both parental phenotypes). Furthermore, a far greater proportion of juvenile animals have EBV with a higher accuracy than can at present be obtained by selection using both parental phenotypes. Thus, reliable information is available on much more of the population than currently exists, which will allow breeders to make more accurate selections earlier in the life of the dog. The accuracy of selection is directly linked to genetic progress, meaning more accurate selection will lead to greater progress in breeding for health. We have demonstrated this to be the case in a wide range of breeds. The broader impact can be realised by noting that the average annual number of registrations of the 15 breeds included in this study, and so that will each have an EBV, is in excess of 80,000, approximately 1/3 of all annual registrations with the UK Kennel Club.

Substantially faster genetic improvement is expected to come via both increased accuracy and greater selection intensity, as the provision of EBV could have a major impact on the ways in which dogs are selected by breeders and pet owners (accompanied by appropriate user information). Currently mate selection is based on ancestral phenotypes and two dogs’ own phenotypes (if known). Using EBV owners of breeding bitches would be able to more accurately assess the genetic merit of potential sires resulting in an improved response to selection, whether phenotypes are available or not. In addition, EBV will be available for all registered animals of the breed, increasing selection intensity opportunities. For example: the projected time to achieve an improvement of 5 points in the median hip score via phenotypic selection, under the guidelines which were in place for the majority of the period covered by the data, range from 30 to over 300 years (NEWF and BEARD respectively) mainly due to weak selection intensity [8]. Although these guidelines have now been amended to promote selection from below the median rather than the mean phenotype, the opportunity to increase selection intensity is more readily presented by EBV (their universality within a breed removing the random sampling of genetic risk from the use of un-scored animals). Selecting breeding stock with EBV below the breed mean is projected to achieve such an improvement in between 9 years for NEWF and 18 years for BEARD. The increases in the proportion of animals with breeding value accuracies greater than that provided by parental phenotypes illustrate that EBV provide, per phenotype, more information on more animals, enabling wider comparison by breeders. An additional benefit from publishing EBV could be the indirect introduction of selection pressure through potential pet owners more accurately differentiating the genetic risk of hip (and elbow) dysplasia among available litters.

It is crucial however that participation in the BVA/KC screening schemes continues – the availability of EBV does not mean scoring is no longer necessary. Phenotypes are the basis of accurate breeding values, and accuracies will rapidly decline if phenotypic information were to become sparse. Theory predicts that EBV accuracy would be expected to increase with participation, and a plot and regression of mean EBV accuracy at birth on the mean annual proportion of sires with hip scores provides empirical support (Additional file 5: Figure S1). Experience in livestock sectors reinforces the theory, where widespread and routine data collection and very large family sizes (i.e. thousands of progeny) can yield EBV accuracies of >0.9, although, it must be noted, accuracies are rarely so high at the time of selection. The resulting message to breeders is simple: continued scoring will maintain and further enhance the accuracy of selection of breeding stock for healthy joints, as well as increasing the pool of animals with reliable information. Moreover, the phenotypic score is of value to breeders and pet owners alike in providing an indicator of not only the genetics but the environmental influence on an individual animal’s hip/elbow joints. While the EBV should guide breeding decisions, the phenotype is useful to inform the appropriate care of the dog that may ameliorate the severity of hip and elbow dysplasia where it occurs.

The accumulation of phenotypes will be particularly critical for future analyses of elbow dysplasia, where the extent of recording is much less than for hip dysplasia, and since elbow score is less heritable than hip score (possibly due in part to the collection of traits described by the elbow score). This study only managed to detect a genetic correlation between hip and elbow scores with enough precision to be statistically significantly different to zero in the LAB. Previously, we demonstrated that bivariate analysis of hip and elbow data can confer significant benefits to the accuracy of EBV for elbow scores, where a favourable genetic correlation exists [17]. Additional elbow score data will be essential to determine more precise genetic correlations between hip and elbow score in BMD, GR, GSD and ROTT, although reported estimates from other studies indicate there may be wide variation across breeds [9,14,16].

While genetic parameters are often (correctly) viewed as specific to each breed, questions can arise as to whether the genetic parameters (h^2^ and r_A_) from one breed may be useful in BLUP analyses (EBV calculation) of another. This is particularly relevant where small population size means that breed-by-breed parameter calculation is not feasible. The analysis of 15 breeds in this study using the same model provided a good opportunity to explore this matter. Results from the meta-analysis indicate that there is more between breed variation in estimates of genetic correlation between hip and elbow score than for heritability of elbow score, across the five breeds for which both traits were analysed. While additional elbow scoring data will therefore be expected to result in more consistent estimates of heritability across breeds as sampling variance is reduced, the estimates of genetic correlation between hip and elbow score are expected to reflect the greater between breed variation in the true parameter. The slightly higher estimate of between breed variance of heritability for hip score compared to that for elbow score may reflect the greater number of breeds included in the analysis for that trait, and inclusion of additional breeds not currently in the sample may prove an outlier to this current collection. Nevertheless, results from the meta-analysis suggest that the heritability of both hip and elbow score are remarkably consistent across breeds, and that most of the observable variation in estimates is due to sampling variation. The across breed estimate of the residual correlation between hip and elbow score is small (with a small s.e., 0.024 ± 0.035), and the meta-analysis revealed only small between breed variation in such estimates (Additional file 6: Table S3). This implies that across breeds there is a large degree of independence in non-genetic environmental risk factors on dysplasia of the hip and elbow joint. This finding across multiple breeds supports an earlier observation on the small environmental correlation between hip and elbow score in LAB [17] and is somewhat surprising given that both dysplasias are developmental orthopaedic diseases.

All breeds included in this study showed an improving genetic trend with respect to hip and elbow score, except the SHUSK, suggesting that phenotypic selection to date has had a small but beneficial impact. The increasing genetic propensity towards hip dysplasia in the SHUSK was matched by the phenotypic trend (regression of total hip score on date of birth showing a yearly rise of 0.075 score points), which has been observed previously [31]. However, the SHUSK had the best hip scores of all the 15 breeds analysed here. It may be that the historical role of the SHUSK as a sled dog has entailed *de facto* selection against lameness, but that increasing popularity as pets or show dogs has weakened this tacit selection pressure. The popularity of the breed in the UK has risen quickly recently, from 829 registered in 2000 to 2,209 in 2010. While the general hip condition of the SHUSK remains better than for many other breeds, breeders should be aware of the detrimental trend. It serves as an example that the transition to a popular pet breed be accompanied by tools, such as EBV, that protect the qualities of the breed for which it is valued.

The results presented here indicate that the GDN has been subject to the greatest selection intensity for reduction in hip score, equivalent to excluding the 18% of animals with the worst hip scores from breeding. This is in line with former breeding guidelines based on the mean hip score and has been accompanied by a phenotypic decline in hip score of over 0.6 points per year (from regression of total hip score on date of birth) and a fall in the mean hip score from 24.35 in 1990 to 14.77 in 2010. The GDN is not a numerous breed, with a mean of 324 dogs registered per year from 2000–2010, but appears to have a large proportion of breeders committed to including health traits in selection objectives; for example over 80% of sires and dams undergo hip scoring. While slightly greater genetic progress was observed in the NEWF, a larger estimate of heritability and shorter generation interval meant that the derived selection intensity was smaller than for the GDN. However across all breeds and traits, regression of genetic gain on the proportion of breeding animals scored did not show significant association (P > 0.05). This demonstrates that quantity of data alone does not guarantee genetic improvement, but that it must be accompanied by the appropriate breeding advice and the motivation by breeders to act upon it. Across comparable breeds, the rates of genetic progress calculated in this study were broadly typical of those that have been previously reported [16].

Substantial improvements in the predicted accuracy of selection, and therefore genetic progress, based on estimating breeding values have been quantifiably demonstrated here for a wide range of breeds, including a number of the more uncommon breeds. For the more uncommon breeds, selection against diseases such as hip dysplasia is more problematic when based on phenotypes alone as there may be only a small number of the candidates with a record, and so making a small breed smaller. Therefore an approach to increasing numbers of candidates with usable information, as demonstrated here, should be welcome. Rarer breeds are more likely to suffer the effects of genetic over-contribution of some animals to future generations, usually through the widespread use of popular sires. Where selection does take place in small populations (which it must do to improve welfare where hip dysplasia is prevalent, as argued in the introduction) a balance must be struck between genetic progress in reducing the burden of disease on the one hand, and minimising the risk of the emergence of a novel genetic disease on the other, which can be measured by the rate of inbreeding. The inbreeding coefficient *per se* was found to be largely unrelated to, and have only a small effect on, hip and elbow score in this study. However, one drawback with the use of EBV based on pedigrees and phenotypes is that they too can promote greater rates of inbreeding in the course of generating more progress [32]. This need not be inevitable, but instead places an emphasis on increasing awareness of inbreeding among breeders, and making more tools available to help them manage rates of inbreeding as EBV are introduced.

In this study we elected to conduct a deterministic prediction of the superiority of EBV accuracy over that of selection using phenotype. An alternative method would be to use simulation. However, simulations are stochastic and can be prone to error in some situations. A further disadvantage of simulation is a lack of insight into the underlying causes, which when encountered through deterministic use of empirical data can then be used in induction. The reported superiority of mean EBV accuracies over the accuracy of selection on individual hip score phenotype reported here were smaller than reported by Malm et al. using simulation [23], however there tended to be fewer animals with phenotypes in our data, implying less information. Comparison of EBV accuracy with selection on parental phenotypes shows the improvement was of similar magnitude.

EBV for hip and elbow dysplasia are routinely computed and published in Norway, Finland and Denmark for up to 38 breeds and in Sweden for 5 breeds (K Maki, personal communication), in Germany for GSD, and in the USA for LAB. The public release of EBV described in this study is anticipated in the UK in 2013. The abundance of EBV for hip and elbow dysplasia in so many countries raises the prospect of the globalisation of scoring and evaluation schemes. Analyses determining the genetic correlations between individual scoring protocols would enable dogs to be evaluated under any (participating) scheme (UK registered dogs evaluated under the FCI scheme and Scandinavian dogs participating in the BVA/KC scheme for example) while still having an EBV in the country of registration [25]. It should be noted, however, that not all scoring protocols may be equal in terms of predicting the lameness associated with hip and elbow dysplasia and consequential OA [33]. To address this further research focussing on identifying OA and lameness later in the life of scored dogs would be welcome. Fortunately, the manner in which EBV for canine health are presented offers an ‘outward continuity’, allowing improvements to be made to the computational model or to the evaluation protocol, as well as the utilisation of international data, without noticeable disruption to the end user [25].

## Conclusion

The use of EBV by dog breeders is projected to facilitate considerable improvements in the response to selection for healthier hip and elbow joints in a wide range of breeds, through both enhanced accuracy and greater abundance of information. Across the 15 breeds analysed here estimates of heritability of hip and elbow score were remarkably consistent, and phenotypic selection has been successful in eliciting genetic progress, albeit very slowly, in all breeds except the SHUSK. However, substantial improvement in the accuracy of selection via use of EBV was demonstrated across all breeds, for both dogs with and without a phenotype. The availability of EBV for hip score for 15 UK registered pedigree dog breeds will provide information on the genetic risk of disease in nearly a third of all dogs annually registered by the UK KC, with in excess of a quarter having an EBV for elbow score as well.

## Competing interests

TWL is fully funded and SCB partly funded by the UK Kennel Club Charitable Trust. The funders had no role in study design, data analysis, decision to publish, or preparation of the manuscript. Hip and elbow score data and pedigree was collated and provided by the UK Kennel Club. JAW declares no competing interests.

## Author contributions

TWL, SCB & JAW conceived and designed the analyses; TWL performed the analyses; TWL & JAW analysed the results; TWL, SCB & JAW wrote the paper; all authors read and approved the final manuscript.

## Supplementary Material

Additional file 1: Table S1 Summary statistics of hip scores of all 15 breeds. Click here for file

Additional file 2: Appendix 1 Derivation of accuracy of breeding values including F. Click here for file

Additional file 3: Appendix 2 Derivation of accuracy of mass (phenotypic) selection. Click here for file

Additional file 4: Table S2 Summary statistics of elbow scores for 5 breeds. Click here for file

Additional file 5: Figure S1 Plot of EBV accuracy on proportion of sires with phenotypes. Click here for file

Additional file 6: Table S3 Summary of meta-analysis. Click here for file
