# Supplementary material for: Comparative analyses of genetic trends and prospects for selection against hip and elbow dysplasia in 15 UK dog breeds
Source: BMC Genet. 2013 Mar 2;14:16. doi: 10.1186/1471-2156-14-16 (PMC3599011; doi:10.1186/1471-2156-14-16)
Supplement: Additional file 1: Table S1 — Summary statistics of hip scores of all 15 breeds. [file 1471-2156-14-16-S1.pdf]

Additional Table 1. Summary statistics of hip scores of all 15 breeds

BREED	ndata	mean	mode	median	SD	skew
AKT	1750	9.74	6	8	8.64	3.82
BEARD	2187	10.63	10	10	6.40	4.31
BORD	4941	12.79	10	11	8.27	3.55
ENG	2133	17.04	10	13	12.64	2.39
FCR	4377	8.54	8	8	5.43	4.59
GDN	1594	20.16	10	14	16.04	1.77
NEWF	3317	23.35	9	14	20.49	1.46
RR	1901	9.85	6	8	8.83	4.11
SHUSK	2721	7.89	8	8	4.38	1.82
TT	2003	12.51	10	10	9.77	4.08
BMD	3372	14.57	8	10	12.65	2.82
GR	22205	16.91	10	12	13.10	2.40
GSD	23765	17.58	10	12	15.64	2.46
LAB	38453	12.39	8	9	11.35	3.59
ROTT	7001	11.04	6	8	9.28	3.61

Number of records (ndata), mean, mode, median, standard deviation (SD) and coefficient of skewness (skew) of hip scores are shown for all 15 breeds. The top panel are the 10 breeds for which only hip score was analysed, while the bottom panel are the 5 breeds for which both hip and elbow score were analysed. Breed abbreviations: Akita [AKT], Bearded Collie [BEARD], Bernese Mountain Dog [BMD], Border Collie [BORD], English Setter [ENG], Flat Coat Retriever [FCR], Gordon Setter [GDN], Golden Retriever [GR], German Shepherd Dog [GSD], Labrador Retriever [LAB], Newfoundland [NEWF], Rottweiler [ROTT], Rhodesian Ridgeback [RR], Siberian Husky [SHUSK] and Tibetan Terrier [TT].
