# Supplementary material for: Comparative analyses of genetic trends and prospects for selection against hip and elbow dysplasia in 15 UK dog breeds
Source: BMC Genet. 2013 Mar 2;14:16. doi: 10.1186/1471-2156-14-16 (PMC3599011; doi:10.1186/1471-2156-14-16)
Supplement: Additional file 2: Appendix 1 — Derivation of accuracy of breeding values including F. [file 1471-2156-14-16-S2.pdf]

APPENDIX 1

Derivation of accuracy of breeding values including F

The accuracy of evaluation (*r*) for a breeding value is the correlation between the true value (*TBV*) and its estimator (*EBV*). A property of an *EBV* calculated by the process of BLUP for a normally distributed random variable is that it is a best linear unbiased estimate. Therefore:

$$TBV = EBV + \epsilon$$

The value of 1 for the slope of the regression of *TBV* on *EBV* is an expression of the unbiased property. The error term ε is the prediction error, which has no covariance with the *EBV*, otherwise the linear prediction of *TBV* by *EBV* could be improved, contradicting *EBV* itself being the best predictor. The *var(ε)* is the prediction error variance or *PEV*. Therefore it follows $var(TBV) = var(EBV) + PEV$, and $cov(TBV, EBV) = var(EBV)$. So following the standard expression for the correlation $r = \sqrt{(var(EBV)/Var(TBV))}$ and $r = \sqrt{(1 - PEV/var(TBV)}$ (for example, see Bijma (2012)). The *PEV* is obtained directly from the ASREML analysis used here, whereas $var(TBV)$ is obtained from the leading diagonal of Wright's Numerator Relationship Matrix (**A**) that is used within the BLUP evaluation. For an animal *i* the leading diagonal of **A** is given by $1+F_i$ where F_i is the inbreeding coefficient of animal *i* with respect to the same base as is used to calculate **A** and $var(TBV) = (1 + F_i)V_A$ where V_A is the additive genetic variance in the base generation which is directly estimated by REML methods. Therefore:

$$r = \sqrt{(1 - PEV_i/[(1 + F_i)V_A])}$$

This is used as an approximation when the assumption of normality fails, as typically the *TBV* is unknown, and empirical estimates are then impossible.
