# Supplementary material for: Comparative analyses of genetic trends and prospects for selection against hip and elbow dysplasia in 15 UK dog breeds
Source: BMC Genet. 2013 Mar 2;14:16. doi: 10.1186/1471-2156-14-16 (PMC3599011; doi:10.1186/1471-2156-14-16)
Supplement: Additional file 3: Appendix 2 — Derivation of accuracy of mass (phenotypic) selection. [file 1471-2156-14-16-S3.pdf]

APPENDIX 2

Derivation of accuracy of mass (phenotypic) selection

The accuracy of selection is the correlation of the predicted breeding value (PBV) and the true breeding value (A). Since accuracy is a correlation it is not affected by linear transformations, so it is assumed that the phenotypes (P) have mean 0 and variance of 1. In phenotypic selection the PBV = h^2P, and since PBV and P are linearly related then selection on one or the other is equivalent. Therefore since $var(P) = 1$, $var(A) = h^2$, and

$$r = cov(A, PBV)/(h\sqrt{var(PBV)}) \quad (1)$$

For mass selection on the individual's phenotype, then $cov(A, PBV) = h^4$ and $\sqrt{var(PBV)} = h^2$, so *r* *r = h*, and this is also shown in Falconer and Mackay (1996).

When the PBV is based on the average of the parental phenotypes, PBV = ½h^2 (P_{sire} + P_{dam}). Then

$$cov(A, PBV) = cov(A, {}^1/_2\ h^2(P_{sire} + P_{dam})) = cov(A, {}^1/_2\ h^2(A_{sire} + A_{dam})) = {}^1/_2\ h^4 \quad (2)$$

which assumes no selection and random mating. The transition from *P* to *A* for the parents in equation (2) is valid because it is a standard assumption that the environmental component of the phenotype has no covariance with the breeding value. Similarly

$$var(PBV) = {}^1/_2\ h^4 \quad (3)$$

leading to the result

$$r = \sqrt{{}^1/_2}\ \ h \quad \approx 0.71\ h \quad (4)$$

With selection there may be changes in heritability between parent and offspring generation caused by LD. In this case the value of h^2 in equations (2) and (3) refer to the parental generation and *h* in equation (1) refers to the offspring generation. With the infinitesimal model these changes are described by the Bulmer effect (Bulmer, 1971), and the heritability in the offspring will be slightly lower than in the parents to a degree that is dependent on the selection intensity. However the heritability asymptotes rapidly towards a steady state over ~ 3 generations, so these effects are assumed negligible here. It is also assumed that the phenotypes have been corrected for nuisance fixed effects, and without such corrections the accuracy will be lower.

Bulmer, M. G. (1971). "The effect of selection on genetic variability." American Naturalist **105**(943): 201-211.

Falconer, D. S. and T. F. C. Mackay (1996). Introduction to Quantitative Genetics, Longman.
