# Supplementary material for: Comparative analyses of genetic trends and prospects for selection against hip and elbow dysplasia in 15 UK dog breeds
Source: BMC Genet. 2013 Mar 2;14:16. doi: 10.1186/1471-2156-14-16 (PMC3599011; doi:10.1186/1471-2156-14-16)
Supplement: Additional file 4: Table S2 — Summary statistics of elbow scores for 5 breeds. [file 1471-2156-14-16-S4.pdf]

Additional Table 2. Summary statistics of elbow scores for 5 breeds

	ndata	mean	mode	median	SD	skew
BMD	1733	0.57	0	0	0.87	1.41
GR	2184	0.22	0	0	0.52	2.70
GSD	1675	0.19	0	0	0.51	3.19
LAB	7456	0.15	0	0	0.46	3.59
ROTT	566	0.61	0	0.5	0.74	0.92

Number of records (ndata), mean, mode, median, standard deviation (SD) and coefficient of skewness (skew) of elbow scores are shown for the 5 relevant breeds. Breed abbreviations: Bernese Mountain Dog [BMD], Golden Retriever [GR], German Shepherd Dog [GSD], Labrador Retriever [LAB], Rottweiler [ROTT].
