# Supplementary material for: Comparative analyses of genetic trends and prospects for selection against hip and elbow dysplasia in 15 UK dog breeds
Source: BMC Genet. 2013 Mar 2;14:16. doi: 10.1186/1471-2156-14-16 (PMC3599011; doi:10.1186/1471-2156-14-16)
Supplement: Additional file 5: Figure S1 — Plot of EBV accuracy on proportion of sires with phenotypes. [file 1471-2156-14-16-S5.pdf]

Additional Figure 1. Plot of EBV accuracy on proportion of sires with phenotypes

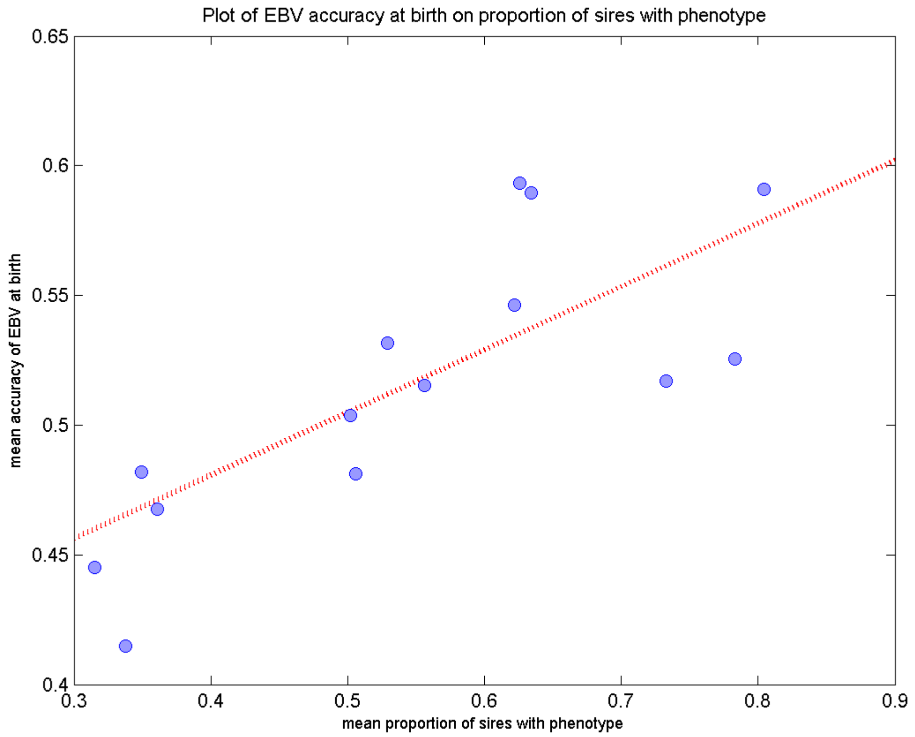

Plot of mean hip score EBV accuracy of all 2011 born dogs (without phenotype) on the mean annual proportion of sires with a phenotype. Regression coefficient = 0.24 (s.e. 0.058), 95% CI 0.13 - 0.36.
