# Supplementary material for: Comparative analyses of genetic trends and prospects for selection against hip and elbow dysplasia in 15 UK dog breeds
Source: BMC Genet. 2013 Mar 2;14:16. doi: 10.1186/1471-2156-14-16 (PMC3599011; doi:10.1186/1471-2156-14-16)
Supplement: Additional file 6: Table S3 — Summary of meta-analysis. [file 1471-2156-14-16-S6.pdf]

Additional Table 3. Summary of meta-analysis.

Parameter	Wt mean	Wt s.e.	v	mu	s.e.
h^2_{hips}	0.367	0.0068	0.0018	0.382	0.0143
h^2_{elbows}	0.212	0.0206	0.0008	0.218	0.0259
r_A	0.246	0.0464	0.0131	0.216	0.0756
r_E	0.017	0.0182	0.0036	0.024	0.0352

The table shows, for parameters heritability (h^2) of and genetic and environmental correlation (r_A and r_E) between hip and elbow score: 1) The weighted mean and standard error of parameter estimate (calculated using the reciprocals of the variance of estimates of all breeds in the sample). 2) The estimate of across breed variance (v). 3) The best estimate of the mean (mu) and s.e. of the parameter for the population of breeds from pooled meta analysis. If v = 0 then mu is identical to Wt mean.
